# T-Cell Receptor Excision Circle/Kappa-Deleting Recombination Excision Circle-Based Newborn Screening Program for Severe Combined Immunodeficiency in Kumamoto, Japan

**DOI:** 10.1007/s12013-025-01873-5

**Published:** 2025-09-02

**Authors:** Yuya Kinoshita, Jun Kido, Takaaki Sawada, Keishin Sugawara, Fumiko Nozaki, Tomoyuki Mizukami, Madoka Nishimura, Shinichiro Yoshida, Ryutaro Tsuru, Kimitoshi Nakamura

**Affiliations:** 1 Department of Pediatrics, Graduate School of Medical Sciences, Kumamoto University, Kumamoto, Japan; 2 Department of Pediatrics, Kumamoto University Hospital, Kumamoto, Japan; 3 Department of Pediatrics, Faculty of Life Sciences, Kumamoto University, Kumamoto, Japan; 4 Center for Clinical Genetics, Kumamoto University Hospital, Kumamoto, Japan; 5 Department of Pediatrics, National Hospital Organization Kumamoto Medical Center, Kumamoto, Japan; 6 KM Biologics Co., Ltd, Kumamoto, Japan; 7 1-1-1, Honjo, Chuo-Ku, Kumamoto City, 860-8556 Japan

**Keywords:** Kappa-deleting recombination excision circle, Newborn screening, Severe combined immunodeficiency, T-cell receptor excision circle

## Abstract

Severe combined immunodeficiency (SCID) is a life-threatening hereditary disorder that requires early diagnosis and intervention. Therefore, this study aimed to evaluate the utility of T-cell receptor excision circle (TREC) and kappa-deleting recombination excision circle (KREC) measurements in newborn screening (NBS) for SCID and other primary immunodeficiencies in Kumamoto Prefecture, Japan. In a TREC-based NBS program (February 2019–March 2022, *N* = 43,658), six newborns (0.014%) underwent immunological testing, and three (0.007%) were diagnosed with T-cell lymphopenia. In a subsequent combined TREC/KREC-based NBS program (April 2022–March 2023, *N* = 12,335), eight newborns (0.065%) required further testing, and one (0.008%) was diagnosed with X-linked agammaglobulinemia. Decreased KREC levels were observed in two newborns exposed to maternal azathioprine (AZP). The TREC levels tended to be lower in more premature infants, whereas the KREC levels showed no clear correlation with gestational age (GA). Both the TREC and KREC levels were reduced in infants with low birth weights (BWs). These findings indicate that the combined TREC/KREC-based NBS program is an effective approach for detecting T- and B-cell immunodeficiencies. However, clinical factors such as maternal AZP exposure, low GA, and low BW may affect TREC and/or KREC levels, necessitating careful interpretation of results from dried blood spot samples.

**Supplementary Information:**

The online version contains supplementary material available at 10.1007/s12013-025-01873-5.

## Introduction

Severe combined immunodeficiency (SCID) is a life-threatening hereditary immunodeficiency disorder that typically occurs during early infancy. Although affected neonates may appear healthy at birth, severe opportunistic infections often develop during early infancy, and failure to thrive is frequently observed. Adverse outcomes, including secondary infections from live vaccines, have also been reported [1].

SCID arises from various genetic mutations, such as defects in the interleukin-2 receptor gamma chain, adenosine deaminase, and recombination-activating genes 1 and 2. These defects primarily result in T-cell dysfunction and, in some cases, may also impair B-cell or natural killer (NK) cell function [2]. Approximately two decades ago, the incidence of SCID was estimated to be about 1 in 100,000 live births [1]. More recently, Wakamatsu et al. identified two patients with SCID among the 137,484 live births in Aichi Prefecture, Japan, between April 2017 and December 2021 [3].

Hematopoietic stem cell transplantation (HSCT) remains the most effective treatment for SCID. Survival is significantly improved when HSCT is performed before the onset of infections, highlighting the critical importance of early diagnosis and intervention [4, 5]. In recent years, gene therapy has emerged as a viable treatment option for X-linked SCID, with improved vector safety compared with previous trials [2]. Newborn screening (NBS) programs for SCID have been introduced in several countries [6–13].

Globally, the T-cell receptor excision circle (TREC)—a circular DNA fragment generated during T-cell receptor gene rearrangement in thymocytes—is widely used as a marker in SCID NBS programs [1]. TREC levels reflect the output of newly formed T lymphocytes, and significantly reduced TREC levels indicate impaired T-cell production. In addition to TREC, the kappa-deleting recombination excision circle (KREC)—a byproduct of immunoglobulin kappa light-chain gene rearrangement in developing B lymphocytes—has been incorporated in some SCID NBS protocols [14].

KREC-based screening facilitates the detection of B lymphocytopenia; however, its application remains controversial due to higher false-positive rates [15].

Since October 2020, the attenuated live rotavirus vaccine has been included in Japan’s routine immunization schedule and is administered to infants between 6 and 14 weeks of age [16]. The administration of attenuated live rotavirus vaccines to patients with SCID may lead to chronic gastroenteritis, resulting in severe stunting and wasting, and poses a significant risk of life-threatening complications despite intensive medical care [17, 18]. Considering this risk, the Japanese government has acknowledged the necessity of incorporating SCID into the NBS program [19, 20] and has announced substantial increases in funding to support NBS expansion and enhance follow-up care [21].

Therefore, this study aimed to evaluate the utility of a TREC-based NBS program conducted between February 2019 and March 2022, followed by a combined TREC/KREC-based program from April 2022 to March 2023. We also aim to present the results of our SCID NBS program and discuss its feasibility and characteristics.

## Materials and Methods

### NBS Program

**Fig. 1:**
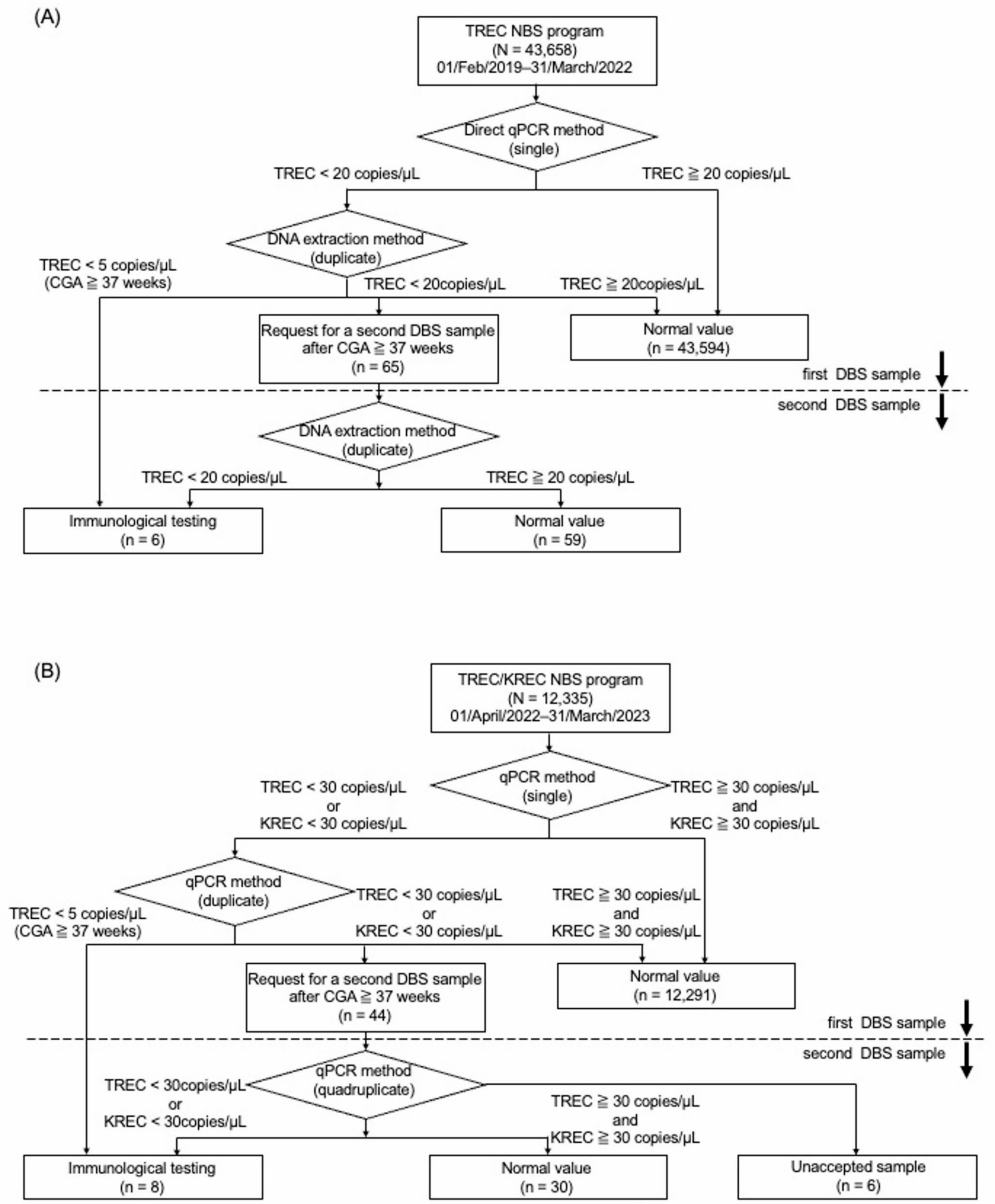
**Summary of the TREC- and TREC/KREC-based NBS programs.** Flowcharts of the (A) TREC- and (B) TREC/KREC-based NBS programs. In the TREC-based NBS program, the TREC cutoff was set at 20 copies/µL. In the TREC/KREC-based NBS program, both the TREC and KREC cutoff values were set at 30 copies/µL. For samples collected from newborns with a corrected gestational age (CGA) of ≥ 37 weeks, the emergency TREC cutoff was set at 5 copies/µL TREC, T-cell receptor excision circle; KREC, kappa-deleting recombination excision circle; NBS, newborn screening; qPCR, quantitative polymerase chain reaction; CGA, corrected gestational age

In 2019, Japan’s NBS program included more than 20 disorders, primarily involving amino acid, organic acid, and fatty acid metabolism disorders, as well as endocrine conditions such as congenital hypothyroidism and congenital adrenal hyperplasia [22, 23]. In this program, blood samples were collected from the heel of newborns between days 4 and 6 of life. As an expanded screening initiative in Kumamoto Prefecture, Japan, TREC- and TREC/KREC-based NBS programs were conducted from February 1, 2019, to March 31, 2022, and from April 1, 2022, to March 31, 2023, respectively. Kumamoto Prefecture has a population of approximately 1.7 million (Fig. 1A–B). Prior to the initiation of the TREC-based NBS program, NBS for lysosomal storage disorders had already been implemented in the prefecture [24–27]. Both the TREC- and TREC/KREC-based NBS programs were performed voluntarily with parental cooperation. Written informed consent was obtained from the parents and legal guardians of all participating newborns. The consent rates were 96.5% for the TREC-based program and 96.1% for the TREC/KREC-based program.

### TREC Measurement

TREC values were measured using dried blood spots (DBSs) collected for routine public mass-screening tests. Discs measuring 1.2 mm in diameter were punched from each DBSs and placed into a 96-well PCR plate (Watson Co., Ltd., Tokyo, Japan). Each well was filled with 20 µL of 0.4 M potassium chloride in phosphate-buffered saline, heated at 50℃ for 3 min, and centrifuged. The supernatant was subsequently discarded, and 20 µL of reaction mixture was added to each well. This mixture contained amplification primers, a reporter dye/quencher-labeled probe, and BIOTAQ™ HS DNA polymerase in Ampdirect^®^ Plus (Shimadzu Co., Kyoto, Japan) (Additional file 1). Quantitative polymerase chain reaction (qPCR) was performed using a QuantStudio^Ⓡ^5 system (Thermo Fisher Scientific K.K., Tokyo, Japan) with the following program: 95℃ for 10 min, followed by 45 cycles of 94℃ for 30 s and 60℃ for 1 min. TREC values were quantified using a TREC copy number standard prepared by a contract laboratory (Nihon Gene Research Laboratories Inc., Sendai, Japan). This qPCR protocol without DNA extraction is referred to as the “direct PCR method.” A preliminary study determined that the cutoff value of 30 copies/µL for TREC was too high; therefore, the threshold was set at 20 copies/µL. If the TREC value obtained using the direct PCR method fell below 20 copies/µL, a 3.2-mm disc was punched from the same DBS, and genomic DNA was extracted using the NucleoSpin Tissue XS kit (TAKARA Bio Inc., Shiga, Japan). The TREC levels were remeasured by qPCR (DNA extraction method). Although the primary cutoff was set at 20 copies/µL, immediate immunological testing was promptly performed if the TREC value was < 5 copies/µL in samples obtained from newborns with a corrected (CGA) of ≥ 37 weeks. This emergency criterion was introduced midway through the program. If the TREC value was < 20 copies/µL, a second DBS sample was requested after the newborn reached a CGA of 37 weeks to reduce false-positive results. Immunological testing was required if the TREC values remained < 20 copies/µL in the second DBS sample.

### TREC/KREC Measurement

**Fig. 2:**
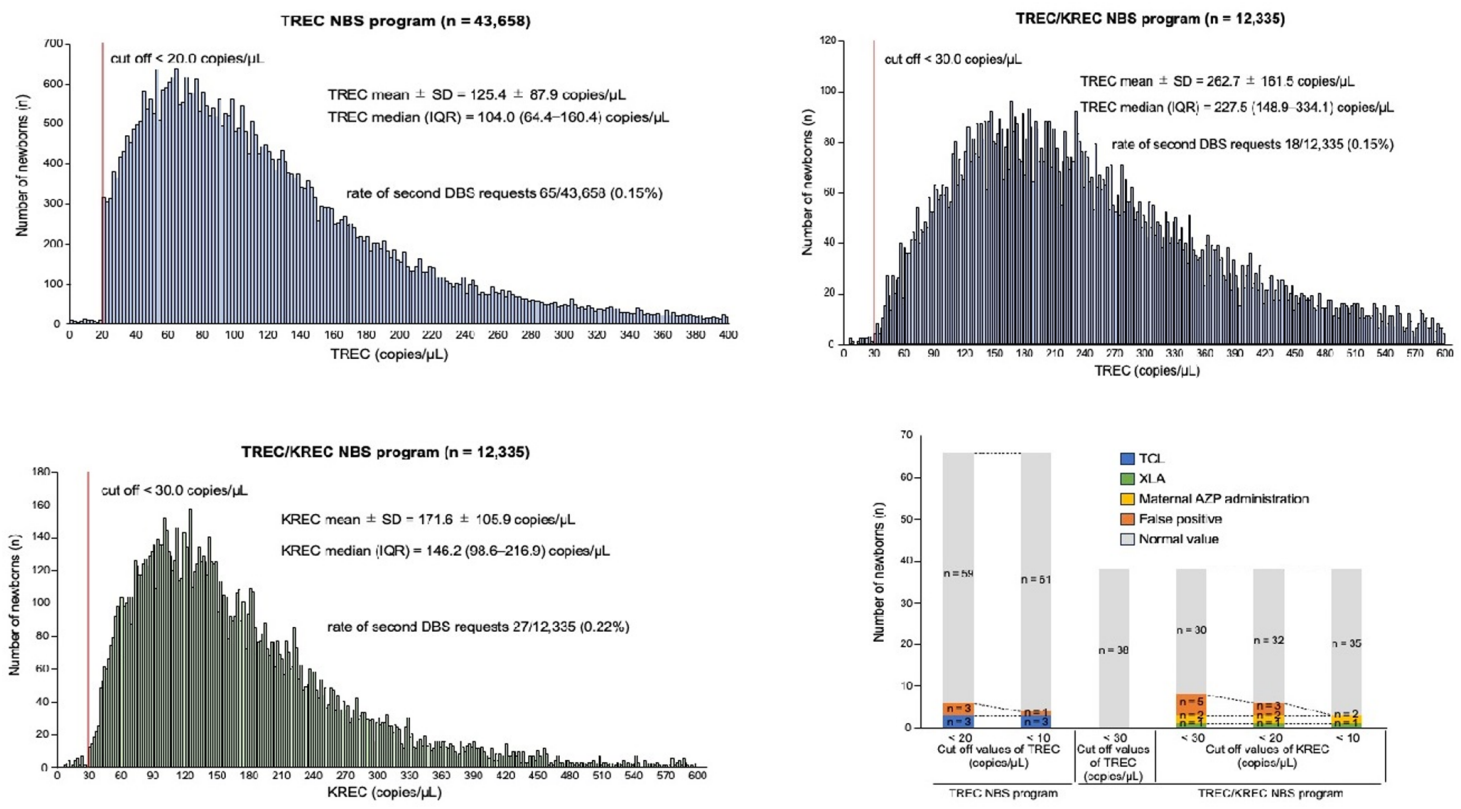
**TREC and KREC distributions and cutoff evaluation in NBS programs.** (A) Histogram of TREC levels in the TREC- and (B) TREC/KREC-based NBS programs. (C) Histogram of KREC levels in the TREC/KREC-based NBS program. (D) Results from the second DBS samples in the TREC- and TREC/KREC-based NBS programs TREC, T-cell receptor excision circle; KREC, kappa-deleting recombination excision circle; NBS, newborn screening; IQR, interquartile range; TCL, T-cell lymphopenia; XLA, X-linked agammaglobulinemia; AZP, azathioprine

TREC and KREC values were similarly measured from DBSs collected for other routine screening tests using mass spectrometry. Discs measuring 1.5 mm in diameter were punched from each DBS, and analysis was performed using the NeoSMAAT TREC/KREC^®^ T/K/S kit (Sekisui Medical Co., Ltd., Tokyo, Japan), which is also applicable for NBS for spinal muscular atrophy [28, 29]. Measurements were initially obtained by qPCR using a single test per sample in accordance with the manufacturer’s instructions. A preliminary study demonstrated that the previous cutoff of 20 copies/µL for TREC corresponded to 30 copies/µL with the new TREC/KREC method, resulting in equivalent second DBS request rates of 0.15%. Accordingly, the TREC cutoff was set at 30 copies/µL (Fig. 2A and B). The initial cutoff for KREC was tentatively set at 30 copies/µL, with adjustments planned during program operation if necessary. If the TREC or KREC value was < 30 copies/µL, qPCR was repeated in duplicate. Immediate immunological testing was performed when the TREC value was < 5 copies/µL in the samples obtained from newborns with a CGA of ≥ 37 weeks. When either TREC or KREC values were < 30 copies/µL, a second DBS sample was requested after the newborn reached a CGA of 37 weeks, and measurements were repeated in quadruplicate to reduce false-positive results. Immunological testing was required if either the TREC or KREC values in the second DBS sample remained < 30 copies/µL.

### Immunological testing

Newborns who met the criteria for immunological testing underwent flow cytometric analysis in addition to routine blood tests. The cluster of differentiation (CD) 3+, CD4+, CD8+, CD19+, CD45+, CD56+, and CD45RA+ naïve T-cells were evaluated. Genetic analyses were performed for patients with T-cell lymphopenia (TCL) or B-cell deficiency (BCD), except in those with known underlying disorders such as 22q11.2 deletion syndrome or secondary causes such as postoperative chylothorax. Initial genetic screening was conducted using targeted next-generation sequencing (NGS) panels for SCID-related genes (20 genes) or BCD genes. If no pathogenic variants were identified, extended panels covering approximately 400 immune-related genes or whole-exome sequencing (WES) were performed.

SCID was defined based on the classification established by the Primary Immune Deficiency Treatment Consortium [30]. TCL was defined as a T-cell count of < 1,500/µL [31]. Complete DiGeorge syndrome (cDGS), a non-SCID primary immunodeficiency, was defined as a condition with a CD3^+^ or naïve T-cell count of < 50/µL resulting from severe thymic hypoplasia or aplasia [32]. BCD was defined as a CD19^+^ cell count of < 2% of the total lymphocytes, and X-linked agammaglobulinemia (XLA) was diagnosed by confirming a *BTK* mutation via genetic testing [33].

### Flow Cytometric Analysis

Flow cytometric analysis was performed using a BD FACSCanto™ Ⅱ flow cytometer (Becton, Dickinson and Company, Franklin Lakes, NJ, USA) to quantify the absolute counts of CD3^+^ T-cells, CD4^+^ T-cells, CD8^+^ T-cells, CD45RA^+^ T-cells (naïve T-cells), CD19^+^ B-cells, and CD56^+^ NK cells. Two reagent kits were used in accordance with the manufacturer’s instructions: the BD Multitest™ 6-Color TBNK Kit (cat. no. 662967; BD Biosciences, San Jose, CA, USA) and BD Multitest™ Anti-Human CD45RA FITC/CD45RO PE/CD3 PerCP/CD4 APC (cat. no. 340751; BD Biosciences, San Jose, CA, USA).

### Statistical Analysis

Statistical significance was evaluated using the Mann–Whitney U test. All analyses were performed using GraphPad Prism (GraphPad Software Inc., San Diego, CA, USA). Box-and-whisker plots were generated in Microsoft Excel, with boxes representing the interquartile range (IQR) between the 25th and 75th percentiles and horizontal lines indicating the median. Whiskers extended to the smallest and largest data points within 1.5 × IQR below the 25th percentile or above the 75th percentile, excluding outliers. Data points beyond the whiskers were considered outliers and were plotted individually. Values reported as < 6.7 copies/µL were assigned a value of 6.7 copies/µL for statistical analyses and plotting.

## Ethics Statements

This study’s was approved by the Ethics Committee of Kumamoto University (approval no. 2018; approval date: August 18, 2015). Written informed consent was obtained from the parents or legal guardians of all participating newborns.

## Results

### Setting of the TREC- and TREC/KREC-based NBS Programs

Histograms of TREC levels from the TREC-based (*N* = 43,658) and TREC/KREC-based (*N* = 12,335) NBS programs were plotted to evaluate TREC distributions (Fig. 2A–C). In the TREC-based NBS program, the TREC level (mean ± standard deviation (SD)) and median (IQR) were 125.4 ± 87.9 and 104.0 (64.4–160.4) copies/µL, respectively. With a TREC cutoff set at 20.0 copies/µL, second blood samples were requested for 65 of 43,658 newborns (0.15%).

In the TREC/KREC-based NBS program, the TREC level (mean ± SD) and median (IQR) were 262.7 ± 161.5 and 227.5 (148.9–334.1) copies/µL, respectively. With a TREC cutoff of 30.0 copies/µL, second DBS samples were requested for 18 of 12,335 newborns (0.15%). Meanwhile, the KREC level (mean ± SD) and median (IQR) were 171.6 ± 105.9 and 146.2 (98.6–216.9) copies/µL, respectively. Using a KREC cutoff of 30.0 copies/µL, second DBS samples were requested for 27 of 12,335 newborns (0.22%). As this program combined TREC and KREC screening, the overall second DBSs were requested for 44 of 12,335 newborns (0.36%). Although the two programs applied different TREC cutoff values owing to methodological differences, the second DBS sampling rates were nearly identical. The median TREC levels in the TREC/KREC-based NBS program were nearly twice as high as those observed in the TREC-based NBS program.

### TREC-based NBS Program

In the TREC-based NBS program (Fig. 1A), 65 newborns (0.15%) exhibited low TREC levels (< 20 copies/µL) in their DBSs. Upon repeat testing using the same DBSs, six newborns (0.014%) continued to show consistently abnormal TREC levels and subsequently underwent immunological evaluation. Of these, three had immunological abnormalities (Table 1).

**Table 1:** Summary of Newborns Who Underwent Immunological Testing as Part of the TREC NBS Program

Patient No.	Gender	Information at the time of initial blood sampling		NBS TREC(copies/µL)		underlying disease	CD3^+^T cell	CD4^+^ T cell	CD8^+^ T cell	Naïve T cell	Immunological diagnosis	Live vaccine	outcome
		**BW (g)**	**CAG (weeks)**	**1st**	**2nd**		**(/µL)**	**(/µL)**	**(/µL)**	**(/µL)**			
T1	Female	2,612	41	15.5	11.7	Miller-Dieker Syndrome	2,395	1,789	537	1,327	no abnormalities	Eligible	Completion of follow-up
T2	Male	2,802	38	2.5	ND	nothing	504	250	135	204	TCL (c.42 C > A, p.C14*, homozygous )	contraindicated	IVIG therapy Oral TMP-SMX therapy
T3	Male	371	23	17.5	15.9	ELBW CLD	2,999	1,891	889	1,233	no abnormalities	eligible	Completion of follow-up
T4	Male	2,482	39	13.3	7.0	nothing	4,339	1,778	2,271	1,197	no abnormalities	eligible	Completion of follow-up
T5	Male	2,388	40	1.7	1.1	DORV VSD PS	319	190	95	97	TCL due to chylothorax	contraindicated	Undergoing treatment at another hospital
T6	Female	2,312	37	ND	-	CHARGE syndrome	8	0	1	0	CHARGE/cDGS (c.2527 C > T, p.R858*, heterozygous)	contraindicated	HSCT

*TREC* T-cell receptor excision circle; *NBS* newborn screening; *BW* body weight; *CAG* corrected gestational age; *ND* no data; *ELBW* extremely low birth weight; *CLD* chronic lung disease; *DORV* double outlet right ventricle; *VSD* ventricular septal defect; *PS* pulmonary stenosis; *TCL* T-cell lymphopenia; *cDGS* complete DiGeorge syndrome; *IVIG* intravenous immunoglobulin; *TMP-SMX* trimethoprim-sulfamethoxazole; *HSCT* hematopoietic stem cell transplantation

- Patient T2 presented with 504 CD3^+^, 250 CD4^+^, 135 CD8^+^, and 204 naïve T-cells/µL, leading to the diagnosis of TCL. A homozygous nonsense mutation in the PTCRA gene (c.42 C > A, p.C14*) was identified through genetic analysis [34]. Live vaccines were contraindicated, and the patient received intravenous immunoglobulin and oral trimethoprim-sulfamethoxazole.
- Patient T5 had an underlying congenital heart disease (CHD), including double-outlet right ventricle, ventricular septal defect, and pulmonary stenosis. Immunological testing revealed 319 CD3^+^, 190 CD4^+^, 95 CD8^+^, and 97 naïve T-cells/µL, confirming TCL. Postoperative chylothorax following CHD surgery was identified as the cause of TCL. Live vaccines were contraindicated, and the patient continued CHD management at another hospital.
- Patient T6 had only 8 CD3^+^, 0 CD4^+^, 1 CD8^+^, and 0 naïve T-cells/µL. The patient presented with CHD, coloboma, choanal atresia, growth retardation, external ear hypoplasia, and hearing loss. Sanger sequencing identified a heterozygous pathogenic variant c.2527 C > T (p.R858*) in *CHD7*, confirming the diagnosis of CHARGE syndrome. Additional findings included hypoparathyroidism, thymic aplasia, and TCL, consistent with cDGS. Live vaccines were contraindicated, and the patient subsequently underwent successful HSCT [35].

### TREC/KREC-based NBS Program

In the TREC/KREC-based NBS program (Fig. 1B), 44 newborns (0.36%) exhibited low TREC (< 30 copies/µL) or KREC (< 30 copies/µL) levels. Repeat testing using the same DBSs identified eight newborns (0.065%) with persistently abnormal KREC levels, who subsequently underwent immunological testing. Among these, one was diagnosed with a BCD (Table 2).

**Table 2:** Summary of Newborns Who Underwent Immunological Testing as Part of the TREC/KREC NBS Program

Patient No.	Gender	Information at the time of initial blood sampling		NBS TREC(copies/µL)		NBS KREC (copies/µL)		underlying disease	CD3^+^T cell	CD4^+^ T cell	CD8^+^ T cell	Naïve T cell	CD19(+) B cell		Immunological diagnosis	Live vaccine	outcome
		**BW (g)**	**CAG (weeks)**	**1st**	**2nd**	**1st**	**2nd**		**(/µL)**	**(/µL)**	**(/µL)**	**(/µL)**	**(/µL)**	**(%)**			
TK1	Female	2,560	38	133.9	171.9	2.9	3.2	Maternal AZP administration for scleroderma	4,444	3,194	909	3,095	1178	19.7	no abnormalities	eligible	Completion of follow-up
TK2	Male	3,482	38	349.5	852.1	4.6	4	nothing	6,045	4,622	1,170	4,493	7	0.1	XLA-MTS (c.201_1800del)	contraindicated	SCIG therapy
TK3	Female	2,668	41	371.7	316.4	16.4	14	nothing	9,204	6,412	2,608	6,284	471	4.1	no abnormalities	eligible	Completion of follow-up
TK4	Male	2,656	39	250.1	214.3	17.1	19.7	nothing	4,973	3,826	1,028	3,776	192	2.9	no abnormalities	eligible	Completion of follow-up
TK5	Male	3,510	40	129.1	200.1	24.3	22.2	nothing	4,319	3,122	1,063	3,010	245	5.1	no abnormalities	eligible	Completion of follow-up
TK6	Male	2,245	36	65.3	112.9	22.4	28.6	21 trisomy	2,945	2,019	889	1,985	102	3.1	no abnormalities	eligible	Completion of follow-up
TK7	Male	3,277	40	20.3	50.6	180.1	13.4	APVR	3,469	1,966	1,430	1,838	637	11.4	no abnormalities	eligible	follow-up
TK8	Female	2,744	40	88.8	112.6	6.9	3.3	Maternal AZP administration for UC	2,770	1,965	729	1,949	695	18.2	no abnormalities	eligible	follow-up

*TREC* T-cell receptor excision circle; *KREC* kappa-deleting recombination excision circle; *NBS* newborn screening; *BW* body weight; *CAG* corrected gestational age; *AZP* azathioprine; *UC* ulcerative colitis; *XLA* X-Linked Agammaglobulinemia; *MTS* Mohr-Tranebjaerg syndrome; *SCIG* subcutaneous immunoglobulin

- Patient TK2 demonstrated a markedly reduced CD19^+^ B-cell count (7/µL, accounting for 0.1% of lymphocytes) with undetectable serum immunoglobulin A (< 2 mg/dL) and M (< 3 mg/dL) levels. Genetic analysis revealed a gross deletion involving BTK and TIMM8A, consistent with XLA and Mohr–Tranebjaerg syndrome. Live vaccines were contraindicated, and the patient received subcutaneous immunoglobulin replacement therapy.
- Among the remaining seven newborns, two were born to two mothers who received azathioprine (AZP) therapy during pregnancy for scleroderma and ulcerative colitis. None of the newborns exhibited BCD, and both demonstrated spontaneous recovery of B-cell ratios over time.

### Re-evaluation of Cutoff Levels in the Second Measurement Using the DNA Extraction Method

Cutoff levels were re-evaluated to assess false-positive rates in the second measurement using the DNA extraction method (Fig. 2D). In the TREC-based NBS program, the false-positive rate was 3/65 (4.62%). This rate would have decreased to 1/65 (1.54%) without missing any TCL cases if the TREC cutoff had been lowered from 20 to 10 copies/µL. In the TREC/KREC-based NBS program, the false-positive rate for TREC was 0/38 (0%). However, the TREC cutoff could not be fully validated as no abnormal TREC levels or TCL cases were detected.

The total false-positive rate for KREC was 5/38 (13.2%). Notably, this system successfully identified a patient with decreased KREC levels associated with maternal AZP administration. If the KREC cutoff had been lowered from 30 to 20 copies/µL or 10 copies/µL, the false-positive rates would have decreased to 3/38 (7.9%) and 0/38 (0%), respectively, without missing any XLA cases and maternal AZP-associated reduced KREC levels.

### Change in TREC and KREC Levels Based on Gestational Age or Birth Weight

**Fig. 3:**
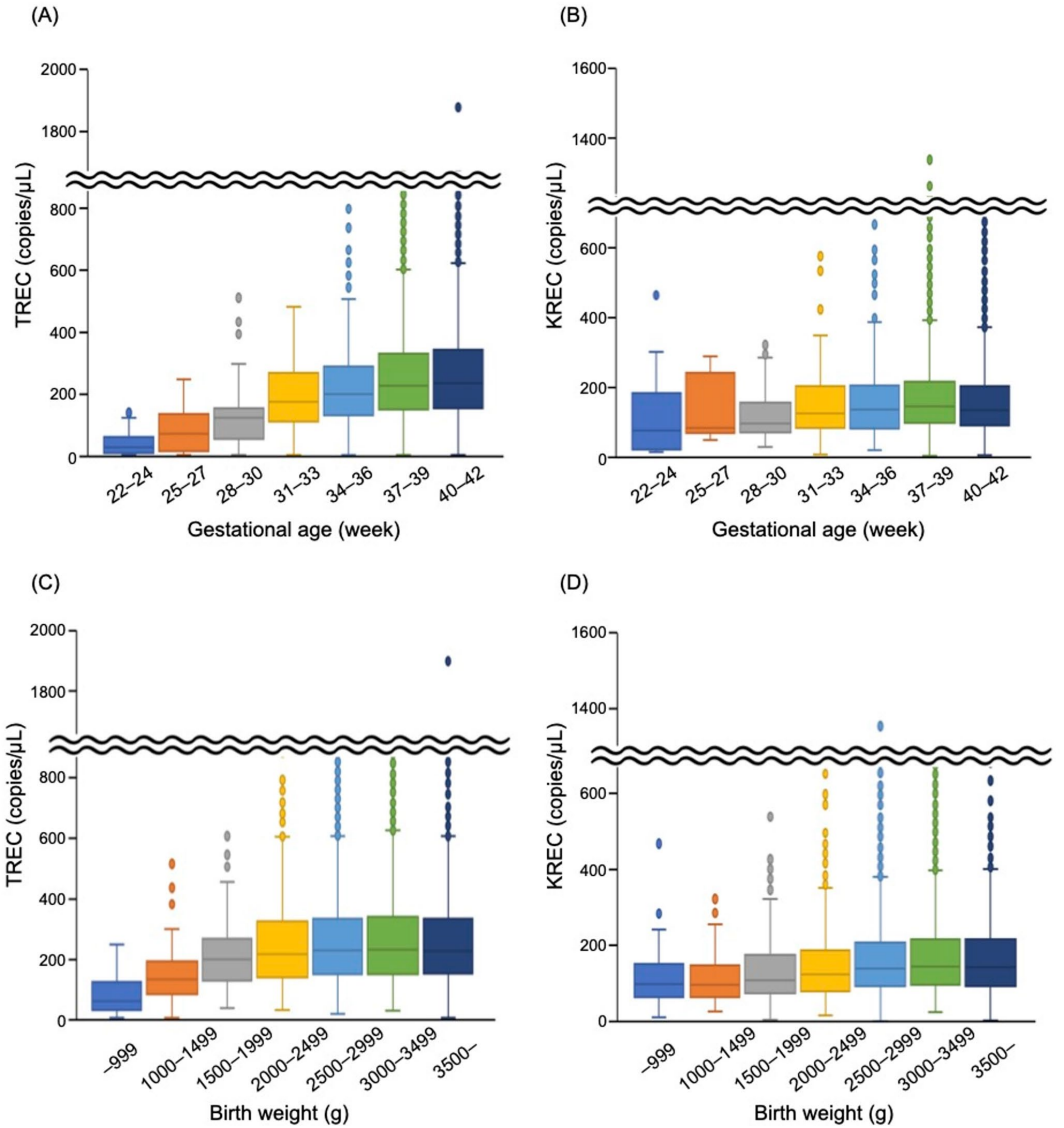
**TREC and KREC levels based on gestational age and birth weight in the NBS program.** Box-and-whisker plots of (A) TREC and (B) KREC levels by gestational age. Box-and-whisker plots of (C) TREC and (D) KREC levels by birth weight TREC, T-cell receptor excision circle; KREC, kappa-deleting recombination excision circle; NBS, newborn screening

The TREC and KREC levels in the TREC/KREC-based NBS program were analyzed based on GA to determine the optimal timing for retesting (Fig. 3A and B). The TREC levels were generally lower in more premature infants, with significant differences (Table 3). By contrast, no clear correlation was identified between KREC levels and GA; however, the KREC levels at a GA of 28–30 weeks were significantly lower than those at a GA of 40–42 weeks. The relationship between TREC/KREC levels and birth weight was also examined (Fig. 3C and D). Both the TREC and KREC levels were generally lower in newborns with low birth weights. Specifically, levels in newborns weighing < 2,000 g were significantly lower than those in newborns weighing > 3,000 g (*p* < 0.001, respectively).

**Table 3:** Results of TREC/KREC NBS Program in Relation to Variables Gestational Age and Birth Weight

	*N*	(%)	TREC (copies/µL)	*p*	KREC (copies/µL)	*p*
Gestational age (weeks)						
22–24	10	0.08	28.95 [12.93–58.95]	< 0.0001	80.20 [35.35–139.30]	n.s.
25–27	15	0.12	69.80 [32.75–134.45]	< 0.0001	88.10 [72.35–221.55]	n.s.
28–30	51	0.41	125.10 [60.60–153.85]	< 0.0001	100.30 [76.55–159.35]	0.0018
31–33	71	0.58	179.70 [123.30–268.40]	< 0.0001	129.30 [92.95–208.90]	n.s.
34–36	414	3.36	200.00 [132.10–290.05]	< 0.0001	141.10 [86.40–211.40]	n.s.
37–39	7357	59.77	227.50 [148.40–331.70]	0.0013	150.90 [102.20–222.00]	< 0.0001
40–42	4390	35.67	234.30 [154.20–344.85]	1	139.80 [95.00–209.15]	1
**Birth weight (g)**						
–999	32	0.26	61.60 [33.10–123.60]	< 0.0001	104.10 [71.73–155.23]	< 0.0001
1000–1499	61	0.50	133.40 [84.30–192.60]	< 0.0001	101.90 [70.50–145.30]	< 0.0001
1500–1999	131	1.06	199.90 [129.35–266.70]	0.0005	114.40 [81.10–181.10]	< 0.0001
2000–2499	779	6.32	217.50 [140.20–324.75]	0.0415	129.70 [85.05–193.70]	< 0.0001
2500–2999	4476	36.34	229.45 [151.50–333.55]	n.s.	144.90 [98.38–214.00]	n.s.
3000–3499	5266	42.75	231.10 [151.30–340.50]	n.s.	151.10 [101.73–222.65]	n.s.
3500–	1573	12.77	227.40 [151.90–333.20]	1	148.90 [99.50–223.20]	1

The p values were calculated by comparing with newborns whose gestational age 40–42 weeks or birth weight 3500 g or more

TREC, T-cell receptor excision circle; KREC, kappa-deleting recombination excision circle; NBS, newborn screening

## Discussion

A TREC-based NBS program and a combined TREC/KREC-based NBS program were implemented as part of the NBS system for the detection of SCID. Accordingly, three newborns with TCL were identified in the TREC-based NBS program, and one with XLA was identified in the TREC/KREC-based NBS program. Live vaccines were contraindicated in four newborns, which enabled the prevention of potentially severe complications and early medical interventions. Similar to SCID, both TCL and XLA require early diagnosis and treatment owing to the high susceptibility of affected individuals to infections. Therefore, the NBS programs proved useful in the early detection and management of these conditions.

The economic advantages of SCID NBS programs, in addition to their clinical benefits, have been reported in other countries [7, 12]. Based on the present findings, SCID NBS programs are both clinically practical and economically beneficial.

The false-positive rates of the screening programs were notably low: 0.007% (3/45,658) and 0.06% (5/12,335) in the TREC-based NBS program and TREC/KREC-based NBS program. One newborn with trisomy 21 (Patient TK6 in Table 2) was identified. Consistent with previous SCID NBS studies, many patients with chromosomal abnormalities—such as trisomy 2—exhibit TREC levels below the cutoff threshold [3, 6]. As illustrated in Fig. 2D, the false-positive rate could potentially be reduced by adjusting the cutoff values for TREC and KREC without overlooking true immunodeficiency cases. However, appropriate adjustment is essential as cutoff levels may vary depending on the assay method. The median TREC levels observed in the TREC/KREC-based NBS program were nearly twice those measured in the TREC-based NBS program. To confirm that this difference was due to assay variability rather than differences in the screened population, TREC levels were compared in an identical set of samples (*n* = 413) analyzed using both assay systems. The average TREC value measured by the TREC-only assay was 149.1 copies/µL, whereas the corresponding value measured by the combined TREC/KREC assay was 239.8 copies/µL. These findings confirm that the observed difference in TREC values is attributable to the differences in the measurement systems rather than variations in the screened population.

In our study, two newborns (TK1 and TK8) exhibited low KREC levels but normal TREC levels due to maternal AZP administration. Two studies reported low KREC levels but normal TREC levels, which normalized upon follow-up, in newborns exposed to maternal AZP [15, 36]. Conversely, other studies have described AZP-exposed newborns with low TREC levels (KREC data unavailable), which subsequently normalized [37, 38]. These findings suggest that maternal AZP exposure during pregnancy may impair fetal TREC and/or KREC production, particularly in fetuses with low thiopurine S-methyltransferase activity—the enzyme responsible for metabolizing AZP [39, 40]. In vitro studies have indicated that B-cells are highly sensitive to AZP, whereas helper T-cells are relatively resistant [41]. This differential sensitivity may account for the observed discrepancies between TREC and KREC levels.

Some previous TREC/KREC-based NBS programs that incorporated KREC measurement did not identify any patient with immunodeficiency based solely on KREC abnormalities [3, 14]. By contrast, the TREC/KREC-based NBS program in Seville (Spain) detected two patients with XLA who had normal TREC levels, and the program in the Czech Republic identified 10 patients with agammaglobulinemia who also had normal TREC levels [36, 42]. In our program, one patient with XLA was diagnosed based on an isolated KREC abnormality despite normal TREC levels. These findings indicate that TREC/KREC measurement can facilitate the early detection of XLA as well as other immunodeficiencies, including non-XLA hypogammaglobulinemia, Nijmegen breakage syndrome, late-onset adenosine deaminase deficiency, and IKZF-related combined immunodeficiency [43–46]. Therefore, the combined evaluation of TREC and KREC represents a valuable approach in NBS programs.

A second DBS sample was requested from infants with a GA of ≥ 37 weeks, in accordance with the previous studies reporting lower TREC levels in preterm neonates [11, 47–50]. Although birth weight was not employed as a criterion for retesting, infants with low birth weight in this study exhibited lower TREC levels, consistent with a previous study [50]. The relationship between KREC levels and GA or birth weight has not yet been fully elucidated. Two studies reported that KREC levels tended to be lower in more premature and low birth weight infants [36, 51]. The present study demonstrated that TREC levels were generally lower in more premature infants and those with low birth weight, whereas KREC levels were significantly lower in infants with low birth weight compared with those with normal birth weight, with no clear correlation between KREC levels and GA. These findings suggest that birth weight may be more closely associated with KREC levels compared with GA.

In conclusion, a large-scale TREC- and TREC/KREC-based NBS program was implemented in Japan, resulting in the identification of three patients with TCL and one with XLA. The TREC/KREC-based NBS program represents a powerful tool for detecting immunodeficiencies affecting T- or B-cell function. However, careful interpretation is warranted when evaluating these biomarkers in newborn DBS samples, as factors such as maternal AZP administration, low GA, and low birth weight may influence TREC and/or KREC copy numbers.

## Supplementary Information

Below is the link to the electronic supplementary material.

Supplementary Material 1

## Data Availability

No datasets were generated or analysed during the current study.
