# Supplementary material for: T-Cell Receptor Excision Circle/Kappa-Deleting Recombination Excision Circle-Based Newborn Screening Program for Severe Combined Immunodeficiency in Kumamoto, Japan
Source: Cell Biochem Biophys. 2025 Sep 2;84(1):549–60. doi: 10.1007/s12013-025-01873-5 (PMC12967461; doi:10.1007/s12013-025-01873-5)
Supplement: Supplementary file 1 — Supplementary Material 1 [file 12013_2025_1873_MOESM1_ESM.pdf]

# PCR primers and probe for the real-time PCR assay

## TREC

```
Forward primer: 5' CCATGCTGACACCTCTGGTT 3'
Reverse primer: 5' TCGTGAGAACGGTGAATGAAG 3'
Probe         : 5' FAM/CACGGTGATGCATAGGCACCTGC/TAMRA 3'
```

## TREC/KREC

NeoSMAAT TREC/KREC kit (Sekisui Medical Co., LTD., Tokyo, Japan) primer and probe sequences are not disclosed.
